# Recent Advances and Future Directions in Downstream Processing of Therapeutic Antibodies

**DOI:** 10.3390/ijms23158663

**Published:** 2022-08-04

**Authors:** Allan Matte

**Affiliations:** Downstream Processing Team, Bioprocess Engineering Department, Human Health Therapeutics Research Center, National Research Council Canada, 6100 Royalmount Avenue, Montreal, QC H4P 2R2, Canada; allan.matte@nrc-cnrc.gc.ca

**Keywords:** downstream processing, monoclonal antibody, process development, continuous bioprocessing, single-use technologies

## Abstract

Despite the advent of many new therapies, therapeutic monoclonal antibodies remain a prominent biologics product, with a market value of billions of dollars annually. A variety of downstream processing technological advances have led to a paradigm shift in how therapeutic antibodies are developed and manufactured. A key driver of change has been the increased adoption of single-use technologies for process development and manufacturing. An early-stage developability assessment of potential lead antibodies, using both in silico and high-throughput experimental approaches, is critical to de-risk development and identify molecules amenable to manufacturing. Both statistical and mechanistic modelling approaches are being increasingly applied to downstream process development, allowing for deeper process understanding of chromatographic unit operations. Given the greater adoption of perfusion processes for antibody production, continuous and semi-continuous downstream processes are being increasingly explored as alternatives to batch processes. As part of the Quality by Design (QbD) paradigm, ever more sophisticated process analytical technologies play a key role in understanding antibody product quality in real-time. We should expect that computational prediction and modelling approaches will continue to be advanced and exploited, given the increasing sophistication and robustness of predictive methods compared to the costs, time, and resources required for experimental studies.

## 1. Introduction

Therapeutic antibodies are complex molecules. Many aspects of their protein chemistry and structure must be considered as part of their design, optimization, development, and manufacturing. An ever-increasing array of antibody formats, fragments, and conjugates are produced in mammalian, insect, or bacterial cells. Isolation of these molecules from product- and process-related impurities such that they possess the correct attributes for their intended therapeutic purpose is the task of downstream processing (DSP). The success of DSP process development and manufacturing is heavily interdependent on the quality of the therapeutic antibody design, including its chemical and structural properties, as well as the upstream process (USP) and associated USP and DSP analytical technologies available to measure product attributes and process parameters.

DSP of therapeutic antibodies continues to undergo revolutionary change. Over the years, DSP has often been described as the rate-limiting bottleneck in therapeutic antibody production. New technologies and paradigms of working have emerged in recent years that are helping to relieve this situation. Pressure continues to increase productivity while decreasing the cost of goods (CoGs). There is also the need for rapid process development of downstream processes for lead therapeutic antibodies to enable a faster path to regulatory submission and commercialization.

The aim of this article has not been to provide a comprehensive review of recent advances in DSP but rather to provide a summary of recent trends and technologies in selected areas, including those that seem of particular importance for therapeutic antibody DSP process development and manufacturing. It is hoped that this review also provides the interested reader a glimpse of where some active subject matter areas within DSP are advancing in the near future.

## 2. Developability Assessment for Lead Antibody Molecules

There is significant attrition of antibody drugs during therapeutic development, with greater than 90% of all molecules failing to advance to commercialization. While a variety of causes have been documented to explain this high attrition rate, including failure to properly identify responsive patient groups in clinical trials, an unacceptable adverse event profile, poor translation of in vivo efficacy obtained in animal models to human hosts, unexpected drug product immunogenicity, and inadequate pharmacokinetic (PK) and pharmacodynamics (PD) profiles, a poor Chemistry, Manufacturing and Controls (CMC) developability profile is also a common cause for failure. While much emphasis for lead selection is often placed on important functional and upstream productivity criteria, a critical assessment of the biochemical and biophysical properties of the expressed product can remain insufficiently addressed, leading to failure in DSP development later.

When developing an engineered protein into a drug substance for manufacture, there is a need to understand the risks, strengths, and weaknesses of the molecule to be developed. Such an analysis will, in the first place, inform if the candidate molecule is truly suitable for investing in DSP development. Parameters that should be evaluated include upstream product titer, clarified harvest and process intermediate stability during potential unit operation hold time ranges, including under low pH, room temperature and freeze-thaw stability conditions, the effect of shear stress or transmembrane pressure (TMP) sensitivity in the context of tangential-flow filtration (TFF) unit operations, and colloidal and conformational stability as a function of product concentration and forced degradation studies in early-stage formulation buffers. It is worth noting that an appropriate suite of analytical assays needs to be established and be made available, without which the necessary measurement of product quality attributes cannot happen. An understanding of these parameters will allow for initial operating limits to be set for DSP unit operation development. These kinds of analyses serve to de-risk process development by identifying molecule liabilities upfront before extensive time, effort and money have been invested in process development.

Several in silico tools have also been developed to assess post-translational modifications (PTMs, reviewed in [1]) or other potential molecule liabilities. The early identification of potentially deleterious sequence or structural features in silico can reduce the overall experimental burden and allows for prioritization of experimental work using often limited resources. PTMs, including aspartic acid isomerization, asparagine, glutamine deamidation, methionine oxidation, lysine glycation, among others, can negatively impact the efficacy, safety, and potency of antibody therapeutics. Such PTMs can occur during production, during DSP unit operations, post-fill-finish, or even post-administration in vivo [2]. In the case of post-translational modifications, predictions that are based on sequence alone appear on average to be less robust than those that also incorporate antibody structural information, either from crystal structures or modelling data, or that in addition utilize molecular dynamics simulations. While in silico prediction methods continue to improve, there are risks with both false positive and false negative results, resulting in the need for at least some degree of further experimental verification. 

Efforts to increase mAb conformational stability through mutation of framework residues can enhance low pH hold stability and reduce aggregation propensity [3]. In this publication [3], a combination of in silico sequence analysis and stability-indicating assays were utilized to identify five sequence sites where mutations increased mAb stability. Statistical sequence analysis has been demonstrated in some cases to be a powerful tool to improve antibody developability properties [3,4,5], even in the absence of obvious structural or sequence-related liabilities. Therefore, it should be considered part of the initial in silico sequence assessment package. 

PTMs which lead to increased heterogeneity in either mAb charge or mass need to be carefully evaluated for their impact. These include C-terminal Lys residues and potential Fab glycans, which can also negatively affect antigen binding or potentially introduce unwanted immunogenicity and the status of intra- and intermolecular disulfide bonds. Intact inter-chain heavy-chain and heavy chain/light chain disulfide bonds are critical for mAb structural integrity, with quantifying mAb free sulfhydryl moieties possible using mass spectrometry-based approaches [6]. This can be particularly important for bi-specific antibodies, where the presence of pairs of distinct heavy and light chains having unique sequences can lead to significant heterogeneity in antibody species if chain pairing is not sufficiently optimized at the design stage. 

Common issues for therapeutic antibody candidates confounding DSP development include a propensity towards aggregation and poor solubility. Both problems can be of particular concern due to the lower pH values typically used for elution in Protein A affinity chromatography as well as for viral inactivation. For this reason, experiments are normally required to establish product stability profiles in the pH 3-4 range as a function of both product concentration and hold time. The use of additives and changes in temperature during low pH hold and subsequent pH adjustment have been explored as potential options to reduce product aggregation [7,8].

While proper assessment of antibody properties, as described above, should certainly contribute to success in DSP, modulation of therapeutic antibody characteristics can also be utilized to enhance protein purification performance directly. This involves protein engineering via sequence modifications to modify mAb properties to improve protein purification outcomes, at least for proof-of-concept evaluation of potential lead candidates. This has been demonstrated, for example, by modifying residues within a mAb heavy chain that result in changes in elution behavior in cation exchange chromatography [9]. A second example is the engineering of amino acid charge differences (EEE/RRR) into two different heavy chain sequences used to construct bi-specific antibodies, facilitating the separation of the desired heterodimers from homodimers post-mixing in vitro [10]. While useful for R&D studies, such non-native sequence modifications, however, run the risk of increasing immunogenicity and anti-drug antibodies. Detailed computational analysis and modelling of the antibodies and fragments thereof (Fab, Fc) can reveal insights into their surface charge properties, which can be used to infer binding strength and orientations with chromatography ligands through QSAR modelling approaches in the case of ion exchange [11] or experimentally using NMR combined with MD simulations with mixed-mode chromatography ligands [12]. These kinds of studies can provide deeper mechanistic insight into molecular level binding between residues of the mAb and the purification resin functional group.

## 3. Adoption of Single-Use Technologies

Historically, durable materials, primarily stainless steel, have been the major material used to construct bioprocessing equipment. There has been an increasing shift towards the utilization of single-use (SU) consumables in recent years, particularly for commercial manufacturing (reviewed in [13]), with the market predicted to represent total sales of USD $33B by 2027 [14]. These consumables include a wide spectrum of products, from bags and liners to filters, pressure and conductivity sensors, flow paths, pump heads, and chromatography columns. More and more process filtration and chromatography skids utilize single-use manifolds and tubing assemblies, often sold by the equipment supplier. Carefully designed and validated SU consumable products takes some of the worries out of executing unit operations, including concerns over bioburden control and the need for execution of cleaning validation procedures. However, the Coronavirus pandemic, has also demonstrated how fragile the supply chain can be for SU consumables and how this can impact timelines for process development and drug product manufacturing. 

There are potential cost and environmental concerns relating to the adoption of SU consumables that need to be considered in terms of how and when they are best utilized. Life cycle analysis studies conclude that SU technologies present an overall lower environmental impact compared to the use of durable systems [15]. Usually, SU technologies are adopted at production scales of less than 2000 L, while at larger volumetric scales, SU costs are higher compared to using durable products and where adaptation of available SU technologies is more complex and presents additional risks. Single-use consumables offer clear benefits to drug manufacturing with regard to safety, the fast turnaround time for batches, and cost savings. While the optimal solution will be case-dependent, in some instances, a hybrid solution consisting of both SU and durable technologies could strike the correct balance between cost, flexibility, and acceptable risk. Smaller, multi-product facilities based on SU technologies offer the potential for increased flexibility and lower costs per batch compared to larger legacy facilities [16]. Such facilities are especially important for the manufacturing of cost-sensitive generic biologics. There are also clear cost advantages when the need for smaller scale SU materials is paired with continuous processing of mAbs, as an alternative to batch production, at either clinical or commercial manufacturing scales [17].

Clarifying upstream productions has historically been a laborious and time-consuming exercise, often utilizing a combination of centrifugation and filtration to process the feedstock. Advances in filtration technology have provided a variety of depth filters with synthetic or natural composition, charged or neutral materials, with various pore sizes and filter areas, depending on the specific application. Depth filter screening has become an important component of process development. An attractive feature of depth filtration is its scalability from the process development lab to GMP manufacturing at various production scales.

The SU bioprocessing product market has grown considerably because of the Coronavirus pandemic, driven by smaller, more flexible, geographically dispersed, multi-use manufacturing facilities and the advent of increasingly small production batches of niche biotherapeutics. One of the future limitations with SU products is the availability of gamma-radiation via ^60^Co sources, one of the primary methods for irradiating sterilization. In this respect, the Bioprocess Systems Alliance (BPSA) has laid out the requirements and risks associated with adopting X-ray irradiation-based sterilization methods [18]. An important element of this change has been to demonstrate that the nature of the radiation source (Gamma vs. X-ray), both of which generate ionized electrons, yields comparable results for material attributes such as extractables [19]. Some suppliers of SU products are actively transitioning through the complex technical and regulatory pathway that will lead to these sterile products soon being commercially available. Due to the potential of ionizing radiation to alter the chemical or physical properties of SU materials, several analytical methods, including Raman [20] and Fourier Transform Infrared (FTIR) [21] spectroscopies as well as Atomic Force Microscopy (AFM) [22] have been applied to evaluate these changes as part of a risk mitigation strategy. Extractable and leachable testing and their risk management are also critical components for the adoption of SU technologies in drug manufacturing [23].

## 4. Continuous Downstream Processing

Traditional batch or fed-batch production processes yield a specific volumetric and mass quantity of product upon harvest and clarification. The product is isolated during the downstream process, which normally consists of a Protein-A chromatography capture step, low pH viral inactivation, one or more polishing chromatography steps, virus nanofiltration, and a UF/DF step to properly formulate the drug substance prior to sterile filtration and bulk filling. Each unit operation is discrete while, at the same time, integrated continuously together into a holistic process workflow (reviewed in [24,25]).

The advent of perfusion-based upstream processes has contributed to developing continuous DSP processes for antibody purification. In a continuous process, the product is constantly removed from the perfusion bioreactor and processed in a series of steps analogous to those used for batch or fed-batch DSP. In a fully continuous DSP process, each step would lead directly into the next in a seamless and automated manner. Facility and technological limitations might prevent the design of a fully continuous DSP process, necessitating some steps to be performed semi-continuously. The case for operating DSP processes in continuous versus batch mode needs to be carefully evaluated with an appropriate risk assessment to make the appropriate decision based on the operational scale, stage of product development, regulatory strategy, and availability of facilities and equipment. Cost of goods modelling for DSP processing using stainless steel, SU, and continuous processes for mAbs produced at various scales has revealed clear cost advantages for continuous processing, in most scenarios, but especially at higher production volume, higher final product mass, and antibody titer [17,26]. A summary of the status of continuous mAb manufacturing adoption for several biotech companies has recently been published [24]. 

For harvesting from perfusion bioreactors, TFF and ATF (alternating tangential flow) filtration are possible. In ATF, alternating pressure and vacuum are applied to the permeate flow using a diaphragm pump. As a result, the reverse flow is used to reduce the fouling of the hollow fiber membrane [27]. Cells and higher molecular weight materials are recirculated back to the bioreactor while the product permeate can be directed towards capture chromatography. ATF devices are available in a variety of scales, from bench to process scale, and in stainless steel or SU formats. 

Other in-line clarification methods have also been developed, including single-pass tangential flow filtration (SPTFF) as well as acoustic wave separation (AWS; reviewed in [24,28]). In SPTFF, the productivity of the separation is enhanced by increasing residence time, either by increasing the filter length or by using multiple parallel short filters. This, thereby, requires only a single pass of the feed stream and no need for retentate recirculation, as is required in conventional batch TFF separations. SPTFF devices, depending on the design, can operate in either UF or DF modes [29] and are commonly employed in DSP unit operations that require process volume reduction. SPTFF may be more appropriate for shear-sensitive molecules, as only a single pass of process fluid through a filter or filter assembly is required. With AWS, a 3D standing sound wave is formed within a chamber where cells clump together at wave nodes prior to sedimentation and removal from the feed stream. Depending on the composition of the feed stream, a secondary clarification step could be required following the AWS primary clarification prior to capture chromatography. An obvious advantage of the AWS is that the mAb product should not be lost during cell separation, as no filter surface is required. Continuous centrifugation could also be considered, although there are limitations in terms of scaling, and legacy facility design could be limiting in terms of practical implementation. As relatively new technologies, the practical utility of SPTFF and AWS as clarification options needs to be established based on details of the specific product and process to which they are to be applied.

The typically high cost of Protein-A affinity resins and the need to underload columns in batch mode at the capture step to minimize product loss are additional factors favoring continuous purification strategies, as the purification productivity of continuous mAb capture is higher compared to batch capture chromatography steps. Continuous chromatography also permits uncoupling of residence time to product capture, allowing for the use of smaller bed volume columns than in batch mode, thereby increasing productivity and reducing costs [24]. Another factor driving continuous purification is the increasing availability of off-the-shelf equipment from a variety of suppliers, ranging from bench-scale purification systems to pilot and process-scale chromatography skids.

Three main continuous chromatographic purification modalities can be described. For example, periodic countercurrent (PCC) systems can consist of three columns, where column 1 is being loaded with any breakthrough captured by a second column (2). Once the first column (1) has been loaded, it can be processed for washing and elution, while columns 2 and 3 continue loading. In such a system, columns are alternately loaded, washed, eluted, and re-equilibrated for the next load cycle. The net result is higher protein purification productivity by continuous cycling of a smaller resin bed volume. Simulated moving bed (SMB) chromatography, which is somewhat more difficult to visualize, consists of solvent moving clockwise and column inlet and outlet valves moving counterclockwise, giving the illusion of a mobile resin matrix (simulated moving bed). A third continuous purification method, designated continuous countercurrent tangential chromatography (CCTC), has also been described. Here, a chromatography resin slurry is pumped countercurrent to successive solvents through hollow fiber (HF) modules which retain the resin while allowing molecules to diffuse through the HF pores. The application of this system to continuous mAb purification has been described [30]. Various adaptations of PCC and SMB exist, with numerous purification systems for various purification scales available from several commercial suppliers. 

Current Protein A resins, available from various suppliers, utilize the Protein A ligand from *Staphylococcus aureus*, which has undergone extensive protein engineering to improve alkaline stability, mAb DBC, and increased mAb elution pH (reviewed in [31]). Engineering strategies include the deletion of accessory protein A domains, the introduction of site-specific residue changes to increase pH stability or increase mAb elution pH behavior, as well as the homo-oligomerization of domains to increase mAb DBC. While generally only small-scale Protein A monoliths or membranes mainly suitable for analytical applications are currently available, electrospun cellulose nanofiber adsorbents (Fibro, Cytiva) coupled to the PrismA affinity ligand have been developed and applied to rapid mAb purification [32]. Experiments comparing purification productivity using MabSelect PrismA resin and Fibro PrismA, both media used in a continuous PCC purification system, revealed greater than 30-fold purified antibody productivity for the nanofiber adsorbent as compared to the bead resin [33].

As with Protein A resins, improvements in commercially available ion exchange and mixed-mode resins have also been made. This resulted in improved pressure-flow characteristics, increased salt tolerance, more uniform bead particle sizes, and enhanced selectivity and binding capacity. In terms of polishing chromatography, both resin-based ion-exchange media, operated in PCC or SMB mode, as well as membrane chromatography devices [34], due to their inherently high convective mass transfer rates, can be employed. A membrane-based chromatography unit operation, often performed in a flow-through mode in part owing to a lower DBC compared to bind-and-elute mode, can also be performed as a SU step, as appropriate. An additional option is adsorbents based on electrospun cellulose fibers having high surface area and porosity, which, like membranes, allow for high mass transfer concurrent with high flow rates. While prototype materials have been reported containing, for example, ion exchange functional groups [35], very few commercial products are currently available.

The viral inactivation step poses logistical difficulties when envisioned within a continuous process. Within batch processes, the product-containing feed stream pH must be lowered to 3.5–4.0 and maintained at this pH for 30–60 min. Then, the pH was readjusted prior to continuing to the next unit operation. While prototype devices, typically plug flow reactors or packed beds, used to perform this operation automatically and continuously have been developed [36,37,38,39], only one commercially-available semi-continuous device is currently marketed [24]. This is an active area for further research and development.

## 5. Mechanistic and Statistical Process Modelling

While experimental approaches, including the application of high-throughput methods to DSP process development, have been historically dominant (reviewed in [40]), modelling methods have and are increasingly being employed as a key tool to gain deep process understanding, reduce time and effort, reduce costs, and increase efficiency. Modelling can serve to focus experimental work where it is most critically needed, may reduce the total amount of experimental effort, and combined, both approaches can increase process knowledge and increase confidence in process robustness. 

There are two main approaches to downstream process modelling, the use of statistical methods, including Design of Experiments (DoE; [41]) and first-principles mechanistic modelling (reviewed in [42,43,44]). Mechanistic models incorporate both mass transport properties within the column as well as protein-resin (adsorption/desorption/diffusion) characteristics, captured as a series of differential equations that quantitatively define time-dependent changes in solute concentration. To utilize these models, some experiments are needed to establish values for mass transfer and resin porosity parameter calibration [45]. Modelling calculations are performed using packages including MATLAB or specific process modelling software, such as the open-source CADET-Process [46] based on CADET [47] or various commercial software products, including Aspen Chromatography™ [48], YPSO-Ionic^®^ [49], and GoSilico™ from Cytiva [50]. There are obvious questions about objectively assessing the quality of a given mechanistic chromatography model, with confidence intervals and scaling behavior [51] or the use of Bayesian inference [52] being proposed as possible solutions. This uncertainty, along with relatively few highly-skilled chromatography modelling personnel being available, has resulted in restricted use of this approach. 

Unlike mechanistic models, statistical methods rely on a mathematical model describing the interactions between factors and responses independently of a physical understanding of the particular system. Consequently, statistical models do not allow for deeper process understanding, a limitation of this approach. Often factors are process-related parameters, while responses represent desired attributes of the product. Not all factors influence responses equally, so it is necessary to identify the most important factors initially using screening designs. Once the main factors have been identified, response surface modelling (RSM) can be used. A variety of commercially available statistical software packages are available with different model options. 

Under those conditions where a particular unit operation does not allow for clear mechanistic understanding, the use of statistical approaches could be considered to be the preferential approach. While it is possible to infer response values for factors in a valid statistical model, it would be inappropriate to extrapolate predictions for factor values that lie outside of the model limits. This is different from a mechanistic model, which, since it is first-principles based, can be used to extrapolate new conditions beyond those used to calibrate the initial model. Derivation of calibration values for mechanistic models typically requires a small number of scale-down bind and elute column chromatography experiments, 5 to 10, depending on what questions are to be addressed using the model. Each new feedstock or chromatography resin would require a new set of model calibration experiments. In statistical models, the number of experiments required can be much larger, depending on the size of the design space, how it is to be sampled, and the number of factors and responses to be measured. Various strategies have been suggested to reduce the number of experiments required and make the workflow more efficient, for example, the use of split DoE designs [53].

Modelling approaches can be used in a variety of downstream development contexts, including process development, process scaling, technology transfer, batch versus continuous unit operations, resin lifetime studies, development of control strategies, and other elements of process validation as well as for root cause analysis of incidents or deviations. The application of mechanistic modelling is most appropriate for column chromatography unit operations, where a well-developed physical understanding exists of the basic thermodynamics, kinetics, and fluid mechanics as applied to such systems. Applications of mechanistic modelling have been extensively described within the literature and include areas such as elution ion-exchange gradient shape and fraction collection strategy to optimize product purity [47], mAb cation-exchange chromatography as a polishing step [51], mAb breakthrough as a function of concentration using a cation exchange membrane absorber, Mustang-S [54] and ionic capacity/ligand density variations in anion exchanger resins [55], as a few examples. 

The further adoption of mechanistic modelling within DSP manufacturing environments should be anticipated as software improves and becomes more available, as the potential increases for real-time analysis with increased computing power. Additionally, it should be anticipated as regulatory authorities accept the role of modelling results as part of the overall manufacturing control strategy and as the user base expands and the level of expertise broadens within the DSP community.

## 6. Process Analytical Technologies

The traditional approach to DSP in-process analytics has involved the collection of samples at the end of various unit operations and submitting these samples for various offline analyses. A more modern approach called Process Analytical Technology (PAT) involves at-line (analysis performed with a sample that is removed from the process stream but is in proximity to the unit operation), on-line (analysis performed using a sample that is diverted from and may be returned to the process stream), or in-line analysis (direct monitoring using a sensor located in the process stream) and sampling. This allows for real-time or near real-time analytical data on product critical quality attributes (CQAs) (reviewed in [56,57,58,59,60]. PAT takes advantage of modern sensors, sampling, and analytical technologies in combination with data management and data mining tools and strategies to gain deeper and more thorough product and process understanding, consistent with QbD expectations. The ultimate goal would represent continuous and comprehensive process and product understanding within the context of the overall process control strategy allowing for manufacturing scenarios such as real-time process control and real-time release testing [61].

PAT methods include several techniques that can be applied to monitor product or process-related parameters for either upstream or downstream unit operations. In DSP, approaches include the use of in-line, durable, or SU sensors to measure conductivity, pH, turbidity, or UV absorbance. The dynamic range for in-line absorbance measurements for protein purification operations can be extended significantly using variable path length UV/vis spectroscopy [62]. Fiber optic surface plasmon resonance (SPR) biosensor probes have been described for on-line measurement of antibody titre [63]. HPLC chromatography-based methods, as an example, are well-established off-line analytical tools used for separation and quantitation of a variety of antibody product attributes, including size and charge variants, glycan profiles, and as a separation interface to mass spectrometry mass-based detection. 

Within the PAT context, various spectroscopic methods used to characterize proteins, including infrared spectroscopy (IR), FTIR spectroscopy, and Raman spectroscopy, have been adopted. Efforts have been made to apply Raman spectroscopy to in-line flow cell monitoring of breakthroughs during Protein A chromatography [64,65,66] and for in-line, real-time antibody concentration estimation in the permeate stream from perfusion bioreactors [67]. Further advancements will be necessary to overcome challenges relating to the weak Raman signal, high sample complexity, and resulting poor signal-to-noise level in the spectra, which currently limit the practical application of this approach for process monitoring and control. In-line light scattering detectors to measure either molar mass by multi-wavelength light scattering (MALS) or size by dynamic light scattering (DLS) provide valuable insight into antibody aggregates and low molecular weight (LMW) species. A combination of in-line HIC-MALS and on-line SEC-MALS (UHP-SEC-µMALS) to identify aggregates during an IgG1 mAb HIC purification has been described [68]. Interestingly in this study, the signal from the in-line MALS detector was used to activate fractionation during HIC purification, allowing for MW-specific fractions to be collected.

The implementation of on-line methods requires sampling from the process stream, either manually or using automated equipment. An on-line process chromatography sampling approach using a 2-position, 6-port valve with a sample loop for analysis of antibody samples by high-performance liquid chromatography (HPLC) cation exchange chromatography (CEX) for quantitation of aggregates and charge variants have been described [69]. In another on-line approach, using a FISP sampling probe and ultra-performance liquid chromatography- process sample manager (UPLC-PSM), PATROL™ system, samples during IEX process chromatography were analyzed by native and denaturing SEC to identify size variants prior to product pooling [70]. In this study, the quantitation of antibody monomer, LMW, and HMW species showed a good correlation between offline and on-line sampling approaches. 

Careful consideration is required for optimal adaptation of PAT for it to have the most impact, based on the specific product, the nature of the DSP process, and the available technology and IT infrastructure. Continuous DSP processing, where there is a constant fluid transfer between unit operations, would seem to benefit naturally from a PAT approach. A recent survey of the BioPhorum Development Group, “PAT monitoring and Control”, representing several biopharmaceutical manufacturers, suggests that PAT implementation has the highest business value for the Protein-A capture and subsequent polishing chromatography steps as compared to other unit operations [71]. Understandably, much emphasis has been placed on incorporating PAT into the Protein-A capture chromatography step, given its process importance and the high resin cost in the context of antibody DSP. An advantage of monitoring the Protein A column, which is typically oversized in batch capture processes to minimize product loss, allows for the detection of product breakthroughs and permits better utilization of column capacity. At-line and on-line SEC analysis for determining mAb purity and formulation buffer excipient concentrations during a UF/DF unit operation has also been described [72], providing a further example of how PAT can be implemented into DSP operations.

Mass spectrometry-based approaches for PAT, primarily bottom-up LC-MS/MS sequence analysis of antibody-derived peptides, have been reported for on-line upstream product monitoring [73]. Designated as a multi-attribute method (MAM), such an approach has been recognized for some time as an opportunity to increase product quality and improve process control during manufacturing. However, at the same time, there is also recognition that practical implementation comes with its own technical and regulatory challenges [74,75]. High-resolution, mass-based detection methods have clear advantages beyond methods that rely on peak detection alone. They can provide amino acid residue-level information on product CQAs and process-related residual host cell proteins. Implementation of both native SEC-MS and Protein-A chromatography-MS strategies, incorporating MS-appropriate LC buffer systems [76], demonstrates the opportunity for utilization of MS approaches in DSP. The incorporation of on-line LC-MS approaches for product monitoring, including 2D-HPLC or capillary electrophoresis (CE) separation-based methods combined with MS detection, represents an exciting, information-rich future direction in DSP enabling PAT development.

## 7. Summary and Conclusions

Many factors, including the need to reduce CoGs, increase manufacturing flexibility as well as reduce and streamline timelines for antibody product development, have driven the availability of new technologies and new ways of thinking about downstream processing. Change has been further accelerated during the Coronavirus pandemic, which at the same time has highlighted the vulnerabilities of supply chains for consumables and equipment and demonstrated the importance of flexible, agile options for process development and manufacturing.

**Figure 1:**
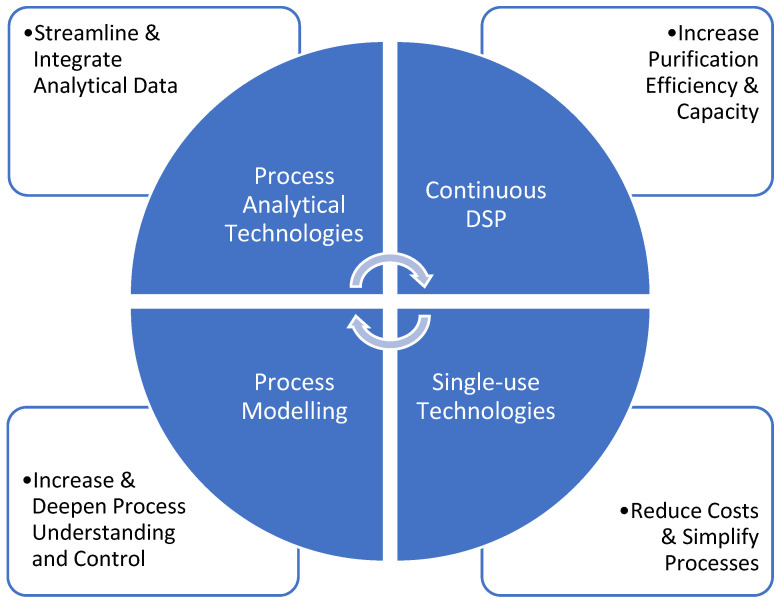
Interrelationship between PAT, continuous DSP, SU technologies and process modelling. For each technology, the primary driver for increasing adaptation is indicated.

There are clear interrelationships between the major technology and process drivers described in this review (Figure 1). SU technologies, for example, play a key role in continuous DSP, where PAT can have an important role in enabling product quality and process control. SU sensors for pH and conductivity that are incorporated into SU tubing assemblies of various DSP equipment enable the required process monitoring while maintaining the simplicity of operation. Process modelling and PAT data management and analysis are deeply intertwined and together can be leveraged to provide deeper process understanding and enhanced process robustness. We should expect further rapid development in DSP-enabling technologies and computational tools that will further streamline process development and manufacturing in the years to come.
